# Fundus Autofluorescence Changes in Age-related Maculopathy

**DOI:** 10.4274/tjo.69260

**Published:** 2018-12-27

**Authors:** Pınar Bingöl Kızıltunç, Figen Şermet

**Affiliations:** 1 Ankara University Faculty of Medicine, Department of Ophthalmology, Ankara, Turkey

**Keywords:** Fundus autofluorescence, lipofuscin, reticular drusen, age-related maculopathy

## Abstract

**Objectives:**

The aim of this study was to describe the fundus autofluorescence (FAF) findings of age-related maculopathy and risk patterns associated with FAF changes.

**Materials and Methods:**

FAF images of 150 eyes with age-related maculopathy were evaluated retrospectively. FAF patterns were classified as normal, minimal change, focal increase, patchy, linear, lace-like, reticular, and speckled pattern. Correlation between patterns and visual acuity, pattern associations at initial visit, and focal atrophy development and pattern alterations during follow-up were evaluated.

**Results:**

At initial examination, 33.3% of the eyes showed no FAF pattern. In the other eyes, the most common patterns were reticular, focal increase, and patchy pattern at rates of 18%, 14.7%, and 11.3%, respectively. There was no correlation between pattern and visual acuity at initial visit. Two coexisting patterns were observed in 4.6% eyes, and the most common pattern in these combinations was reticular pattern (85.7%). Pattern alterations were observed in 5.3% of the eyes during follow-up. Half of these alterations involved transformation to reticular pattern or addition of reticular pattern to the initial pattern. In addition, 13.3% of the eyes developed focal atrophy during follow-up. Development of focal atrophy was more common with focal increase and reticular pattern, with rates of 45% and 30%, respectively.

**Conclusion:**

Presence of reticular pattern may be a risk factor for change and progression of FAF findings in age-related maculopathy.

## Introduction

Age-related macular degeneration (AMD) is a multifactorial degenerative macular disease that causes severe visual loss in elderly patients in industrialized countries.^1,2^ Although the exact mechanism of AMD is unclear, accumulation of lipofuscin and alteration of retinal pigment epithelial (RPE) cells play a role in the early stages of the disease. Focal hyperpigmentation, hypopigmentation, and drusen are the main findings of early AMD. Eventually all retinal layers are affected, especially the RPE and photoreceptor layers.^3,4,5^

Fundus autofluorescence (FAF) imaging is one of the important imaging methods for understanding the pathophysiology and establishing the progression of early AMD, because of the lipofuscin accumulation. Recent studies have described FAF changes in early AMD patients.^6,7^ These FAF changes are important to identify eyes at risk of progression to late AMD.

This study was conducted to describe FAF findings in early AMD and evaluate FAF changes in early AMD stage.

## Materials and Methods

A total of 150 eyes with early AMD were evaluated. Eyes with at least 2 FAF images taken 6 months apart and no accompanying retinal pathology were included in this study. The records and FAF images of the eyes were retrospectively reviewed. Ethical approval was obtained according to the Ankara University Faculty of Medicine Ethics Committee of Clinical Research (number: 10-403-13). The study was conducted in adherence to the Declaration of Helsinki.

FAF imaging was performed using a confocal scanning laser ophthalmoscope; the Heidelberg Retinal Angiography 2 (Heidelberg Engineering, Heidelberg, Germany). Short-wave autofluorescence images were recorded at a wavelength of 488 nm via ≥500 nm barrier filter.

FAF patterns were classified as normal, minimal change, focal increase, patchy, linear, lace-like, reticular, and speckled according to the classification of International Fundus Autofluorescence Classification Group.^7^

It is advised to use at least two imaging methods for the detection of reticular pseudodrusen.^8,9^ Therefore, reticular pseudodrusen was identified according to the presence of reticular pattern on FAF image and spectral-domain optical coherence tomography (SD-OCT). On FAF images, this pattern was identified as an isoautofluorescent area surrounded by halos of reduced autofluorescence. On SD-OCT it was identified as accumulation of hyperreflective deposits in the subretinal space between the RPE and the junction between the photoreceptor inner and outer segments.

FAF patterns at initial visit and during follow-up were evaluated. Eyes exhibiting two patterns at the same time were classified according to the dominant pattern. The relationship between coexisting patterns was investigated. Pattern changes observed during the follow-up period were recorded. In addition, the association between patterns and presence or development of focal atrophy was evaluated. Correlation between patterns and visual acuity at initial visit was investigated.

### Statistical Analysis

All analyses were conducted with the SPSS 15.0 software package (SPSS Inc., Chicago, IL, USA). Kolmogorov-Smirnov test was used to test t he distribution pattern of continuous variables. The results of normally distributed variables were presented as mean ± standard deviation and abnormally distributed variables were presented as median (minimum-maximum). Categorical variables were presented as number and percentage (%). Independent samples t-test was used to compare the means of two independent groups, and Mann-Whitney U test was used to compare medians. When comparing more than two groups, ANOVA analysis was used for means and Kruskal-Wallis test for medians. Spearman’s correlation or Pearson correlation analysis was performed to evaluate relationship between continuous variables according to distribution pattern. P values less than 0.05 were considered statistically significant.

## Results

**Figure 1:**
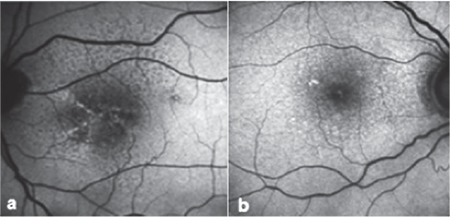
Eyes with two patterns: a) Linear and reticular pattern, b) focal increase and reticular pattern

**Figure 2:**
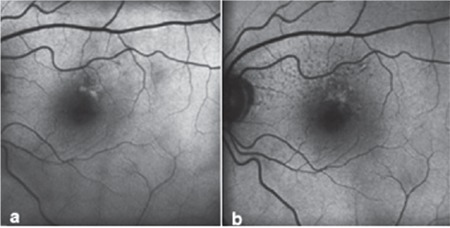
Pattern alteration in fundus autofluorescence imaging: a) Patchy pattern, b) addition of reticular pattern to patchy pattern 64 months later

**Figure 3:**
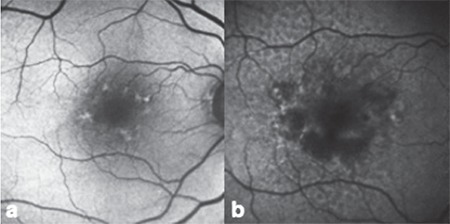
Atrophy development in fundus autofluorescence imaging: a) Linear pattern, b) atrophy and reticular pattern development at 50 months

A total of 150 eyes were evaluated. Mean follow-up period was 42.3 (6-156) months. Mean age of the patients was 75 (59-93) years. The frequency of patterns at initial FAF imaging is shown in Table 1. Fifty eyes (33.3%) had normal FAF imaging in the first examination. The most common patterns were reticular, focal increase, and patchy pattern with the rates of 18%, 14.7%, and 11.3%, respectively. Visual acuities of eyes according to pattern are shown in Table 2. There was no correlation between patterns and visual acuities at initial visit (p=0.073). At initial examination, 4.6% of eyes had two patterns simultaneously and reticular pattern was the most frequent among these pattern combinations (85.7%). Eyes with two patterns are presented in Table 3 and Figure 1. In these eyes, the dominant pattern was accepted as the main pattern. Coexistence of reticular pattern in patients with another pattern was statistically significant (p<0.001). During the mean follow-up period, the baseline pattern changed in 5.3% of eyes (Table 4, Figure 2). Transformation to reticular pattern or addition of reticular pattern to the initial pattern was seen in 50% of the altered patterns. This value was statistically significant (p<0.001). Furthermore, focal atrophy developed in 13.3% of the eyes during follow-up (Figure 3). Development of focal atrophy was significantly more common with focal increase and reticular pattern, with rates of 45% and 30%, respectively (p<0.001) (Table 5).

## Discussion

As our knowledge about the role of lipofuscin accumulation in AMD pathogenesis has increased, FAF has become a more frequently used imaging method for the diagnosis and follow-up of the disease.

Signal alterations shown by FAF imaging do not always correlate with fundus examination findings. Normal fundoscopic findings, drusen, or hyperpigmentation can all be seen in the presence of hyperfluorescence in FAF imaging. This can be explained by the accumulation of different fluorophores located in different retinal cell layers. Delori et al.^6^ and Lois et al.^10^ evaluated FAF findings in eyes without geographic atrophy and choroidal neovascular membrane and showed that FAF alterations are independent of RPE cell alterations and drusen accumulation. They also showed that alterations of FAF signals are independent of fluorescein angiography (FA) and clinical examination findings. They reported that only foveal large and soft drusen (drusenoid RPE detachment) cause FAF alterations. Kellner et al.^11^ showed that in eyes with large drusen (≥125 µm), the main FAF alteration is spots of increased autofluorescence, and that these patients may also exhibit spots of reduced autofluorescence and lines of increased autofluorescence. In addition to detecting different signal alterations in different fundus findings, FAF imaging can also detect changes in areas that appear normal in fundus examination. Therefore, FAF imaging gives more information about the severity and progression of disease in the early stage. All these findings suggest the evaluation of clinical examination and FA findings together with FAF images.

The presence of drusen is a diagnostic criterion for early AMD and also gives information about disease progression. According to the type of drusen, progression to wet AMD can be predicted.^12^ Presence of reticular drusen, a variant of soft drusen, is thought to be a risk factor for late AMD.^13,14,15^ Previous studies showed that progression to late AMD is more frequent in eyes with reticular drusen than in those without.^16,17^ Reticular drusen was also found to be a risk factor for early AMD changes. The Alienor Study showed that the presence of reticular pseudodrusen was significantly associated with an increased risk for early AMD and also that reticular pseudodrusen frequently accompany other signs of early AMD.^18^

Even though fundus examination findings provide information about the progression of AMD, evaluating alterations in lipofuscin accumulation, which plays a role the main mechanism of AMD pathogenesis, has importance in the detection of disease progression. Several studies have demonstrated an association between FAF patterns and progression to late AMD^19,20,21^ and reticular pattern was identified as the high-risk pattern for disease progression. Although the association between patterns and progression to late AMD was evaluated in various studies, there is less information about the relationship between patterns and early AMD.^16,18,22,23^

In our study we observed similar patterns to those described in the International Fundus Autofluorescence Classification Group study.^7^ Bindewald et al.^7^ evaluated FAF patterns in early AMD patients and the most common pattern was speckled pattern (26%) followed by patchy pattern (23%). The frequency of reticular pattern was 15% in their study. In our study, 33.3% of eyes had normal FAF findings. The most common patterns were reticular (18%), focal increase (14.7%), and patchy (11.3%) patterns. In 2 eyes (1.3%) we observed autofluorescence changes that could not be classified according to the International Fundus Autofluorescence Classification scheme. Different studies identified different patterns such as focal confluent,^24^ focal plaque-like,^25^ and scattered.^24^ These patterns’ diversity and the undetermined patterns in our study may be due to the clinical variability of AMD. In addition, the prevalence of patterns varies between studies,^7,26,27^ which may be attributed to regional variation of AMD patterns.

In early AMD, multiple patterns may be seen at the same time, some patterns may disappear, or a new pattern may emerge in addition to the initial pattern over the course of follow-up. These pattern alterations may give new information about disease progression in the early stage. In our study, we evaluated these pattern alterations and found that reticular pattern was most commonly associated with pattern alterations; 4.6% of eyes had 2 coexisting patterns and 85.7% of these were reticular pattern. We also evaluated the pattern alterations during follow-up, and observed pattern conversion or emergence of a new pattern in 5.3% of eyes, with reticular pattern being the most common. Therefore, reticular pattern is not only associated with late AMD but also early AMD changes. These findings may be helpful for recognizing disease progression in early AMD.

In our study we showed that focal atrophy developed in 13.3% of eyes in the follow-up period. Development of focal atrophy was significantly more common with focal increase (45%) and reticular pattern (30%). Focal atrophy may have an importance in the progression to late AMD.

## Conclusion

Presence of reticular pattern in eyes with pattern alterations and focal atrophy development suggest that reticular pattern is also a risky pattern for early AMD progression. Therefore, eyes with reticular pattern should be followed more frequently.

## Figures and Tables

**Table 1:**
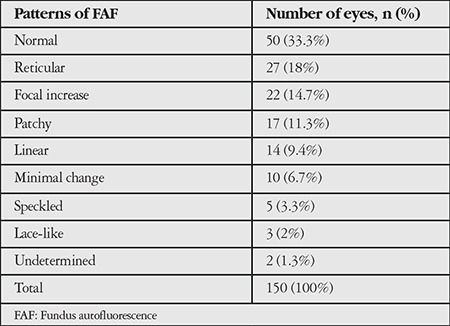
Frequency of patterns at initial fundus autofluorescence imaging

**Table 2:**
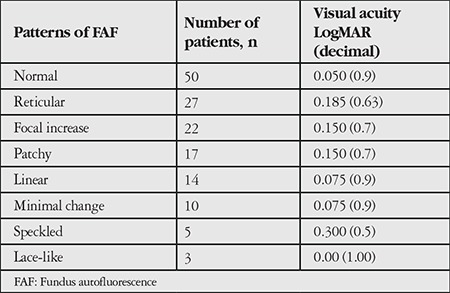
Visual acuities of eyes according to pattern

**Table 3:**
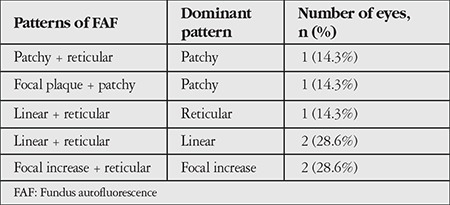
Eyes with two patterns

**Table 4:**
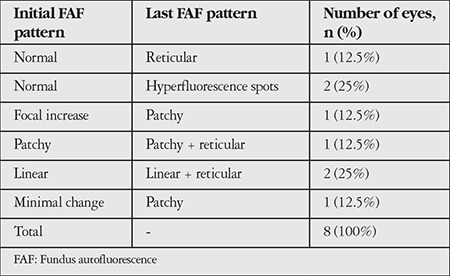
Pattern alterations observed during follow-up

**Table 5:**
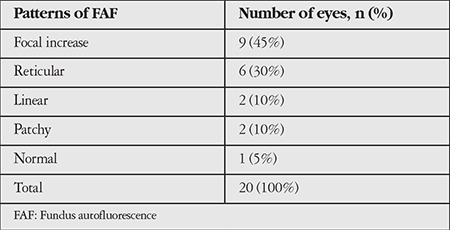
Patterns that developed focal atrophy
